# Parcellation‐based tractographic modeling of the dorsal attention network

**DOI:** 10.1002/brb3.1365

**Published:** 2019-09-19

**Authors:** Parker G. Allan, Robert G. Briggs, Andrew K. Conner, Christen M. O'Neal, Phillip A. Bonney, Brian D. Maxwell, Cordell M. Baker, Joshua D. Burks, Goksel Sali, Chad A. Glenn, Michael E. Sughrue

**Affiliations:** ^1^ Department of Neurosurgery University of Oklahoma Health Science Center Oklahoma City Oklahoma; ^2^ Department of Neurosurgery University of Southern California Miami Florida; ^3^ Department of Neurosurgery Miami Miller School of Medicine Los Angeles California; ^4^ Center for Minimally Invasive Neurosurgery Prince of Wales Private Hospital Sydney NSW Australia

**Keywords:** anatomy, attention, parcellation, tractography

## Abstract

**Introduction:**

The dorsal attention network (DAN) is an important mediator of goal‐directed attentional processing. Multiple cortical areas, such as the frontal eye fields, intraparietal sulcus, superior parietal lobule, and visual cortex, have been linked in this processing. However, knowledge of network connectivity has been devoid of structural specificity.

**Methods:**

Using attention‐related task‐based fMRI studies, an anatomic likelihood estimation (ALE) of the DAN was generated. Regions of interest corresponding to the cortical parcellation scheme previously published under the Human Connectome Project were co‐registered onto the ALE in MNI coordinate space and visually assessed for inclusion in the network. DSI‐based fiber tractography was performed to determine the structural connections between relevant cortical areas comprising the network.

**Results:**

Twelve cortical regions were found to be part of the DAN: 6a, 7AM, 7PC, AIP, FEF, LIPd, LIPv, MST, MT, PH, V4t, VIP. All regions demonstrated consistent u‐shaped interconnections between adjacent parcellations. The superior longitudinal fasciculus connects the frontal, parietal, and occipital areas of the network.

**Conclusions:**

We present a tractographic model of the DAN. This model comprises parcellations within the frontal, parietal, and occipital cortices principally linked through the superior longitudinal fasciculus. Future studies may refine this model with the ultimate goal of clinical application.

## Graphical abstract

Using a meta‐analytic fMRI technique in conjunction with DSI‐based fiber tractography, we describe a tractographic model of the dorsal attention network based on the cortical parcellation scheme previously published under the Human Connectome Project.

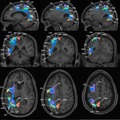

## 1 INTRODUCTION

With advances in neuroimaging techniques, clinicians, and scientists now know that the cerebrum is composed of complex neural networks (Beckmann, De Luca, Devlin, & Smith, 2005; De Luca, Beckmann, De Stefano, Matthews, & Smith, 2006; Thirion, Dodel, & Poline, 2006). Two particular networks, the dorsal and ventral attention networks, have been described in the literature (Chica, Bartolomeo, & Lupianez, 2013; Corbetta & Shulman, 2002). While neurosurgeons can typically preserve primary cortical functions by sparing the primary visual and motor cortices during brain surgery, preservation of higher cognitive networks has proven more difficult (Burks et al., 2017). Therefore, it is likely that advances in brain tumor surgery can be made by improving understanding of network connectivity.

Recent studies have characterized the cortical and subcortical inputs of the dorsal attention network (DAN), which has been described as a bilateral cortical network (Joseph, Fricker, & Keehn, 2015; Shulman et al., 2010), comprising the frontal eye fields, intraparietal sulcus, superior parietal lobule, and visual cortex (Corbetta & Shulman, 2002, 2011; Joseph et al., 2015; Szczepanski, Pinsk, Douglas, Kastner, & Saalmann, 2013). While important, existing descriptions of the DAN lack tractographic detail, limiting our understanding of the underlying structural connections of the network. Advances in human neuroimaging through task‐based functional magnetic resonance imaging (fMRI) have made it possible to study the DAN in greater functional detail (Alnaes et al., 2015; Benedek et al., 2016; Braga, Fu, Seemungal, Wise, & Leech, 2016; Burton, Sinclair, & McLaren, 2008; Dombert, Kuhns, Mengotti, Fink, & Vossel, 2016; Heinen, Feredoes, Ruff, & Driver, 2017; Kato et al., 2001; Kincade, Abrams, Astafiev, Shulman, & Corbetta, 2005; Li et al., 2012; Lyu, Hu, Wei, Zhang, & Talhelm, 2015; Mayer, Dorflinger, Rao, & Seidenberg, 2004; Natale, Marzi, Girelli, Pavone, & Pollmann, 2006; Ozaki, 2011; Sridharan, Levitin, Chafe, Berger, & Menon, 2007). In addition, newly published parcellated brain maps allow us to study network anatomy using a standard cortical atlas and nomenclature (Glasser et al., 2016).

In this study, we constructed a model of the DAN based on the cortical parcellation scheme previously published under the Human Connectome Project (HCP; Glasser et al., 2016). Using relevant task‐based fMRI studies and BrainMap (http://www.brainmap.org/), a collection of open‐access software programs used to generate activation likelihood estimations from fMRI data, we identified the cortical areas involved in the DAN. After identifying the relevant cortical regions of interest, we performed DSI‐based fiber tractography to determine the structural connections between parcellations of the network. Our goal is to provide a more detailed model of structural connectivity of the DAN for use in the future studies.

## 2 METHODS

### 2.1 Literature search

We initially searched for relevant task‐based fMRI studies related to the DAN in BrainMap Sleuth 2.4 (Fox et al., 2005; Fox & Lancaster, 2002; Laird, Lancaster, & Fox, 2005). No research articles were identified using this software. We subsequently queried PubMed on July 12, 2017, for fMRI studies relevant to the network. We used the following search algorithm: “dorsal attention network OR DAN OR goal‐directed attention network AND fMRI.” Studies were included in our analysis if they fulfilled the following search criteria: (a) peer‐reviewed publication, (b) task‐based fMRI study related to the dorsal attention network and/or goal‐directed attentional processing, (c) based on whole‐brain, voxel‐wise imaging, (d) including standardized coordinate‐based results in the Talairach or Montreal Neuroimaging Institute (MNI) coordinate space, and (e) including at least one healthy human control cohort. Only coordinates from healthy subjects were utilized in our analysis. Overall, fifteen papers met criteria for inclusion in this study (Alnaes et al., 2015; Benedek et al., 2016; Braga et al., 2016; Burton et al., 2008; Corbetta, Kincade, & Shulman, 2002; Dombert et al., 2016; Heinen et al., 2017; Kato et al., 2001; Kincade et al., 2005; Liu, Kong, Jin, & Li, 2014; Lyu et al., 2015; Mayer et al., 2004; Natale et al., 2006; Ozaki, 2011; Sridharan et al., 2007). The details of these studies are summarized in Table 1.

**Table 1:** Studies used to generate the activation likelihood estimation of the dorsal attention network

Study	Task	Number of participants	Study coordinate space	Coordinates used in the meta‐analysis		
				*x*	*y*	*z*
Alnaes et al. (2015)	Multiple object tracking	37	MNI	−14	−80	−6
				18	−90	14
				12	−66	34
				−34	−42	−12
				24	−34	−14
				−28	−64	46
				34	−54	40
				20	−56	60
				−20	−56	60
				36	−20	4
				−40	−36	12
				−6	56	−4
				42	30	22
				28	−6	56
				16	2	2
				26	−48	−30
				−6	−80	−24
				−10	−54	12
Benedek et al. (2016)	Anagram and sentence generation	32	MNI	−45	−74	−7
				−20	−63	56
				26	−56	53
				47	−67	−4
Braga et al. (2016)	Saccade distractor task	20	MNI	44	2	54
				−34	0	44
				56	22	28
				−46	4	52
				−56	−34	26
				−50	−22	12
				−54	−56	4
Burton et al. (2008)	Cued vibrotactile stimuli	12	Talarach	−48	−19	35
				−51	−21	43
				−54	−27	19
				−39	−14	17
				−57	−12	14
				−55	−52	26
				−54	−42	3
				−44	−52	42
				−26	−63	48
				−48	−10	40
				−43	−1	36
				−24	−9	57
				−30	−12	51
				−8	12	45
				−8	−7	56
				−39	22	35
				−34	8	10
				49	−27	25
				54	−11	17
				54	−37	37
				50	−37	5
				40	−47	45
				25	−61	50
				35	−2	46
				6	12	47
				5	−8	54
				45	12	24
				38	30	30
				31	17	8
Corbetta et al. (2002)	Cued visual orienting	13	MNI	−31	−55	−16
				35	−57	−20
				−27	−65	−14
				35	−67	−12
				−45	−69	−2
				45	−69	−4
				−31	−83	0
				27	−87	0
				−27	−75	26
				29	−71	22
				−25	−57	46
				−25	−67	48
				27	−59	52
				21	−65	52
				51	−55	4
				−49	−3	46
				39	−9	56
				−23	−11	50
				25	−13	50
				−9	−1	54
				7	3	52
Dombert et al. (2016)	Cued spatial/feature orienting	24	MNI	*Valid spatial orienting*		
				22	6	6
				−22	2	8
				−8	0	58
				30	−2	52
				−26	−8	52
				54	8	38
				−52	2	44
				−22	12	−2
				−52	−24	46
				30	−52	54
				−30	−52	54
				24	−60	52
				−26	−58	58
				48	−72	0
				−44	−72	0
				30	−54	−24
				−38	−62	−28
				*Valid feature orienting*		
				24	8	−4
				−22	4	8
				−6	6	54
				30	−2	50
				−24	−8	52
				46	2	32
				−48	2	36
				−44	−2	10
				−10	−16	8
				−54	−20	26
				33	−52	54
				−30	−52	54
				22	−62	54
				−22	−62	58
				32	−72	26
				−28	−26	24
				30	−54	−24
				−38	−62	−28
Heinen et al. (2017)	Spatial attention shifting task	16	MNI	20	−66	54
				−14	−64	56
				−40	−40	40
				−28	−6	48
				4	−56	44
				36	−40	40
				50	6	34
				−10	−48	52
				32	−6	60
				−28	−74	22
				−30	−50	46
				−46	4	26
				4	8	50
				34	−50	44
				52	−32	40
				−58	−34	34
				34	−76	22
				58	−36	26
				36	20	8
				−22	8	−6
				24	12	−2
Kato et al. (2001)	Cued visual orienting	6	Talarach	44	−42	48
				36	−52	49
				−44	21	27
Kincade et al. (2005)	Cued visual orienting	20	Talarach	−33	−86	−1
				−36	−67	−11
				−43	−72	1
				33	−84	1
				40	−67	−10
				37	−76	−6
				−16	−93	8
				3	−83	13
				−27	−59	34
				31	−61	33
				−38	−50	46
				36	−49	49
				30	−50	39
				25	−51	49
				−19	−60	52
				16	−63	47
				−7	−78	25
				−1	−78	43
				8	−69	28
				10	−73	37
				5	−49	50
				−47	−5	37
				−36	−5	35
				44	−11	44
				−29	−4	49
				−26	−12	54
				38	−11	54
				33	−15	40
				11	−16	60
				34	47	−4
				−33	−84	−5
				−38	−68	−10
				33	−84	1
				38	−69	−7
				−14	−92	10
				14	−90	8
				46	−43	−19
				−62	−53	−11
				33	−63	35
				−38	−50	50
				32	−50	53
				38	−50	42
				26	−45	44
				−23	−57	54
				−13	−59	51
				−6	−79	25
				−1	−78	45
				2	−49	48
				−28	−4	48
				−2	−16	55
Liu et al. (2014)	Visually cued attention	11	MNI	−24	6	51
				27	12	51
				−33	−57	42
				39	−51	39
				33	42	36
				57	18	21
				39	−63	33
				−3	33	18
				3	33	18
				−21	−33	0
				24	−33	−3
				−36	−81	27
				39	−72	33
				30	−54	−3
				−15	−30	6
				15	−27	6
				−54	−15	−12
				42	−63	0
Lyu et al. (2015)	Multiple identity tracking	19	MNI	−18	10	67
				30	15	27
				−31	−56	58
				36	−59	55
				6	−72	0
Mayer et al. (2004)	Cued visual orienting	12	Talarach	−23	−80	19
				28	−51	39
				54	−51	28
				−38	−56	26
				50	−43	15
				46	−63	9
				40	−8	45
				35	−76	16
				−46	−69	7
				−42	−71	−5
				2	−76	36
Natale et al. (2006)	Cued visual orienting	7	Talarach	−17	−76	−3
				25	−82	24
				13	−76	−6
				−2	−82	5
Ozaki (2011)	Cued visual orienting	6	Talarach	31	−5	53
				9	−57	53
				7	4	46
				34	18	12
				18	−66	−11
				1	−12	9
				−27	−8	55
				−12	−60	52
				−2	−3	46
				−30	36	39
				−35	5	14
				−1	−12	9
				−20	−68	−11
				−36	−55	−15
				−53	−58	14
Sridharan et al. (2007)	Passive listening	18	MNI	30	24	−8
				38	46	30
				64	−46	12
				4	34	44
				−46	−26	6
				10	−12	8
				0	−48	44
				−16	−80	−36

### 2.2 Creation of 3D regions of interest

In the original HCP study, parcellation data were studied in CIFTI file format. CIFTI files involve a surface‐based coordinate system, termed greyordinates, which localizes regions of interest (ROIs) on inflated brains (Van Essen & Glasser, 2016). This is in contrast to traditional file formats, such as NIFTI, which denote regions based on volumetric dimensions (Larobina & Murino, 2014). As a result, it was difficult to perform deterministic fiber tractography using ROIs in CIFTI file format. To convert parcellation files to volumetric coordinates, the relevant greyordinate parcellation fields were standardized to the three‐dimensional volumetric working spaces of DSI Studio (Carnegie Mellon, http://dsi-studio.labsolver.org) using structural imaging data available through the HCP. This operation was performed using the Connectome Workbench command line interface (Van Essen Laboratory, Washington University 2016). A single, volumetric ROI was generated for the parcellations identified in the original HCP study (Glasser et al., 2016).

### 2.3 Anatomic likelihood estimation generation and identification of relevant cortical regions

We used BrainMap GingerALE 2.3.6 to extract the relevant fMRI data from the aforementioned studies to create an activation likelihood estimation (ALE) (Eickhoff, Bzdok, Laird, Kurth, & Fox, 2012; Eickhoff et al., 2009; Turkeltaub et al., 2012). All Talairach coordinates identified during literature review were converted to the MNI coordinate space using SPM Conversion in GingerALE. We subsequently performed a single study analysis using cluster‐level interference in the MNI coordinate space (cluster level of .05, threshold permutations of 1,000, uncorrected *p*‐value of .001). The ALE coordinate data were displayed on an MNI‐normalized template brain using the Multi‐image Analysis GUI (Mango) 4.0.1 (http://ric.uthscsa.edu/mango). The preconstructed ROIs of the parcellations were then overlaid on the ALE and compared visually for inclusion in the network.

### 2.4 Network tractography

Publicly available imaging data from the Human Connectome Project was obtained for this study from the HCP database (http://humanconnectome.org, release Q3). Diffusion imaging with corresponding T1‐weighted images from 25 healthy, unrelated subjects were analyzed during fiber tracking analysis (Subjects IDs: 100307, 103414, 105115, 110411, 111312, 113619, 115320, 117112, 118730, 118932, 100408, 115320, 116524, 118730, 123925, 148335, 148840, 151526, 160123, 178950, 188347, 192540, 212318, 366446, 756055). A multishell diffusion scheme was used, and the *b*‐values were 990, 1,985, and 1,980 s/mm^2^. Each *b*‐value was sampled in 90 directions. The in‐plane resolution was 1.25 mm. The diffusion data were reconstructed using generalized *q*‐sampling imaging with a diffusion sampling length ratio of 1.25 (Yeh, Wedeen, & Tseng, 2010).

All brains were registered to the Montreal Neurologic Institute (MNI) coordinate space (Evans et al., 1992), wherein imaging is warped to fit a standardized brain model comparison between subjects (Evans et al., 1992). Tractography was performed in DSI Studio (Carnegie Mellon, http://dsi-studio.labsolver.org) using a region of interest approach to initiate fiber tracking from a user‐defined seed region (Martino et al., 2013). A two‐ROI‐approach was used to isolate tracts (Kamali, Sair, Radmanesh, & Hasan, 2014).

Voxels within each ROI were automatically traced with a maximum angular threshold of 45 degrees. When a voxel was approached with no tract direction or a direction change in greater than 45 degrees, the tract was halted. Tractography was terminated after reaching a maximum length of 800 mm. In some instances, exclusion ROIs were placed to exclude obvious spurious tracts that were not involved in the white matter pathway of interest.

### 2.5 Measuring connection strength

To quantify the strength of the connections identified within the DAN across all subjects, the tracking parameters used within DSI Studio were modified such that the program would count the total number of tracts between any two ROIs based on a random seed count of 2.5 million. Working sequentially through ROI pairs in the network, the number of tracts between regions was recorded for each of the 25 subjects after fiber tractography was terminated under these conditions. The strengths of the connections within the DAN were calculated by averaging the number of tracts between each ROI pair of the network across all subjects.

## 3 RESULTS

### 3.1 Anatomic likelihood estimation regions and their corresponding parcellations

**Figure 1:**
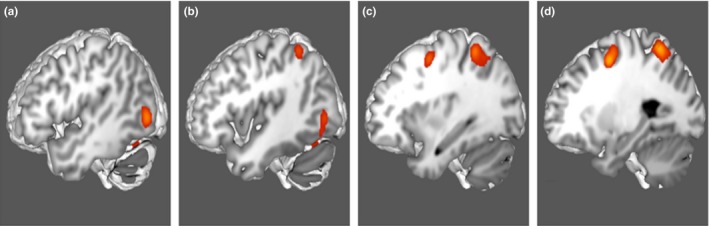
Activation likelihood estimation (ALE) of 15 task‐based fMRI experiments related to goal‐oriented attentional processing. The three‐dimensional ALE data (in red) are displayed in Mango on a brain normalized to the MNI coordinate space. (a–b) ALE data highlighting the left lateral occipital lobe. (b–c) ALE data highlighting the left superior parietal lobule and intraparietal sulcus. (c–d) ALE data highlighting the left frontal eye field region

**Figure 2:**
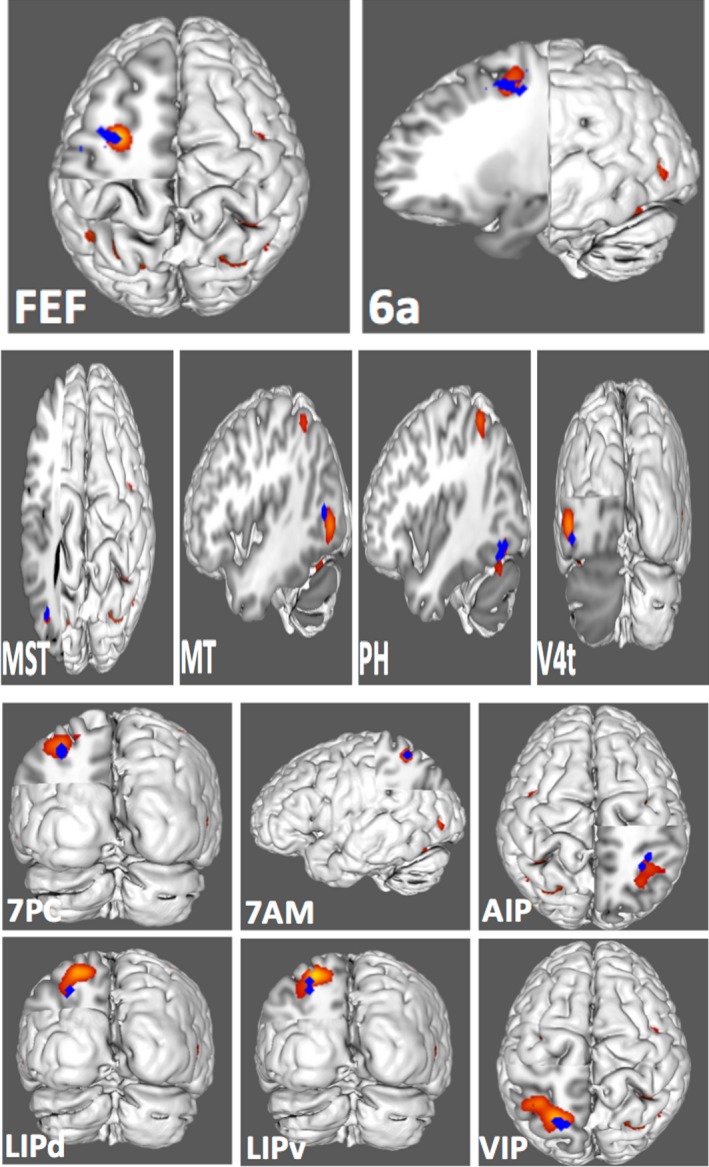
Comparison overlays between cortical parcellations (shown in blue) and the activation likelihood estimation (shown in red) as seen on a left cerebral hemisphere. Regions were visually assessed for inclusion in the network if they overlapped with the activation likelihood estimation. Cortical parcellations assessed for inclusion in our model of the dorsal attention network included areas FEF and 6a in the frontal lobe; areas MST, MT, PH, and V4t in the lateral occipital lobe; and areas 7PC, 7AM, AIP, LIPd, LIPv, and VIP in the superior parietal lobule and intraparietal sulcus. Labels indicate the region of interest shown in each panel

Figure 1 demonstrates the ALE of the 14 DAN‐related, task‐based fMRI experiments included in our meta‐analysis. Highlighted areas include the frontal eye fields, intraparietal sulcus, superior parietal lobule, and visual cortex. Twelve regions of interest were found to overlap the fMRI data, including 6a, 7AM, 7PC, AIP, FEF, LIPd, LIPv, MST, MT, PH, V4t, and VIP. Comparison overlays between these cortical regions and the ALE are shown in Figure 2.

### 3.2 Structural connections within the dorsal attention network

**Figure 3:**
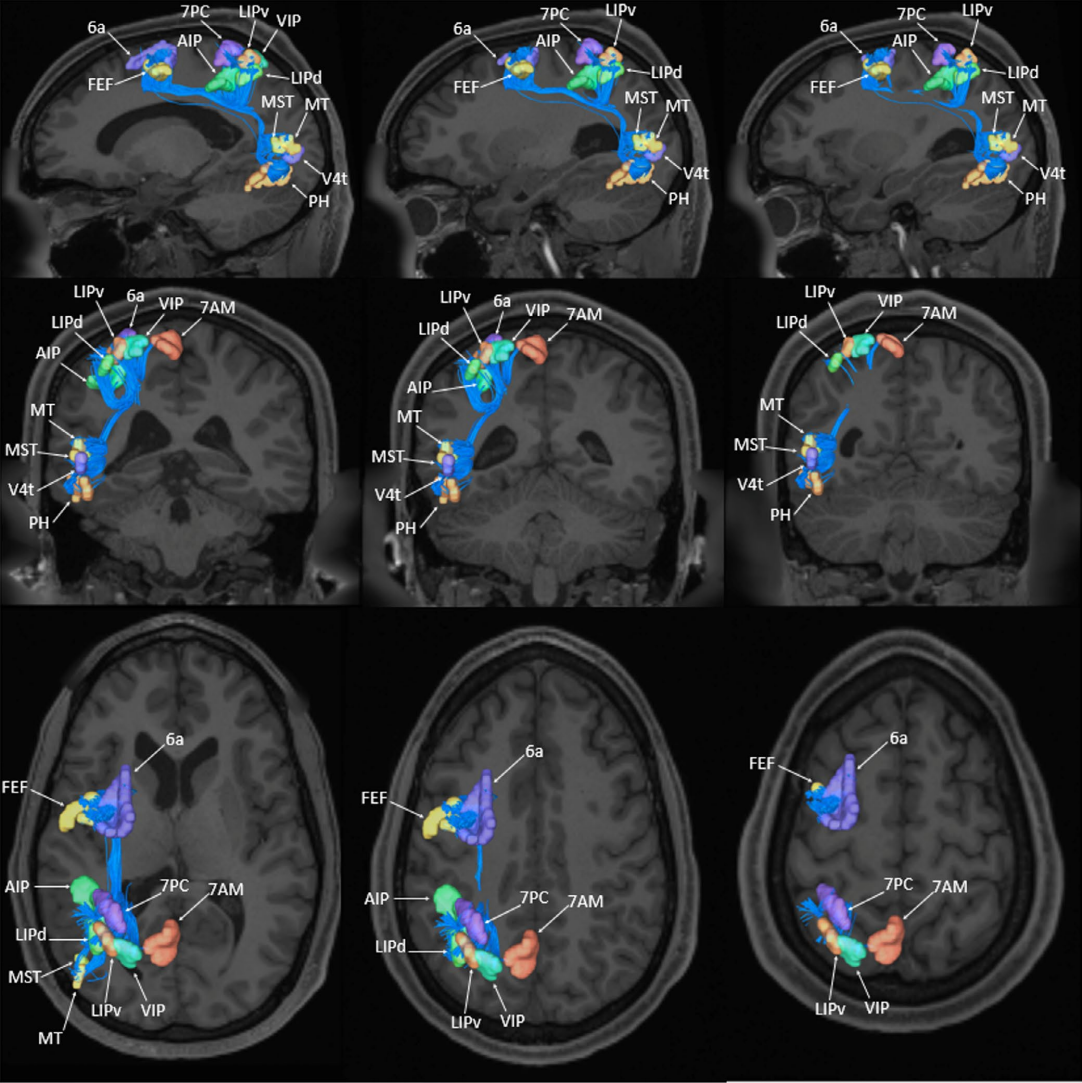
Tractographic model of the dorsal attention network (DAN) as shown on T1‐weighted magnetic resonance images in the left cerebral hemisphere. TOP ROW: sagittal sections through the network demonstrate the extent of the superior longitudinal fasciculus (SLF) which projects between the frontal, parietal, and occipital regions of the DAN. MIDDLE ROW: coronal sections highlight the parieto‐occipital projections of the SLF within the DAN. BOTTOM ROW: axial sections highlight the fronto‐parietal projections of the SLF within the DAN

Deterministic tractography was utilized to show the basic structural connectivity of the DAN. These results are shown in Figure 3. Individual connections within this network are presented in Table 1 which tabulates the strengths of individual connections and lists the type‐specific white matter connections identified between regions.

The cortical areas comprising the DAN can be classified based on the lobe of the brain to which they localize: the frontal lobe (6a, FEF), the parietal lobe (7AM, 7 PC, AIP, LIPd, LIPv, VIP), and the occipital lobe (MST, MT, PH, V4t). U‐shaped fibers form a majority of the connections between ROI pairs within the network. These fibers generally have the same morphology, arising within one part of the cortex before curving 180 degrees to terminate in a part of the brain immediately adjacent to their origin. These U‐shaped fibers represent the local connections between frontal, parietal, and occipital areas.

The superior longitudinal fasciculus (SLF) connects multiple cortical areas of the DAN. The SLF projects between frontal, parietal, and occipital areas of the network as it courses within the subcortical white matter around the Sylvian fissure (Figure 3). In general, connections of the SLF within the DAN can be divided into three subtypes: fronto‐parietal, parieto‐occipital, and fronto‐occipital connections. The fronto‐parietal connections arise from areas 6a and FEF. These fibers initially course inferiorly into the deep white matter of the posterior frontal lobe before curving 90 degrees to continue in the anterior–posterior direction. The fibers pass deep to the sensorimotor cortices before curving 90 degrees superiorly to terminate in the intraparietal sulcus. Area 6a has two connections to regions 7AM and LIPd, and area FEF has connections to areas 7PC, AIP, LIPd, LIPv, and VIP.

**Figure 4:**
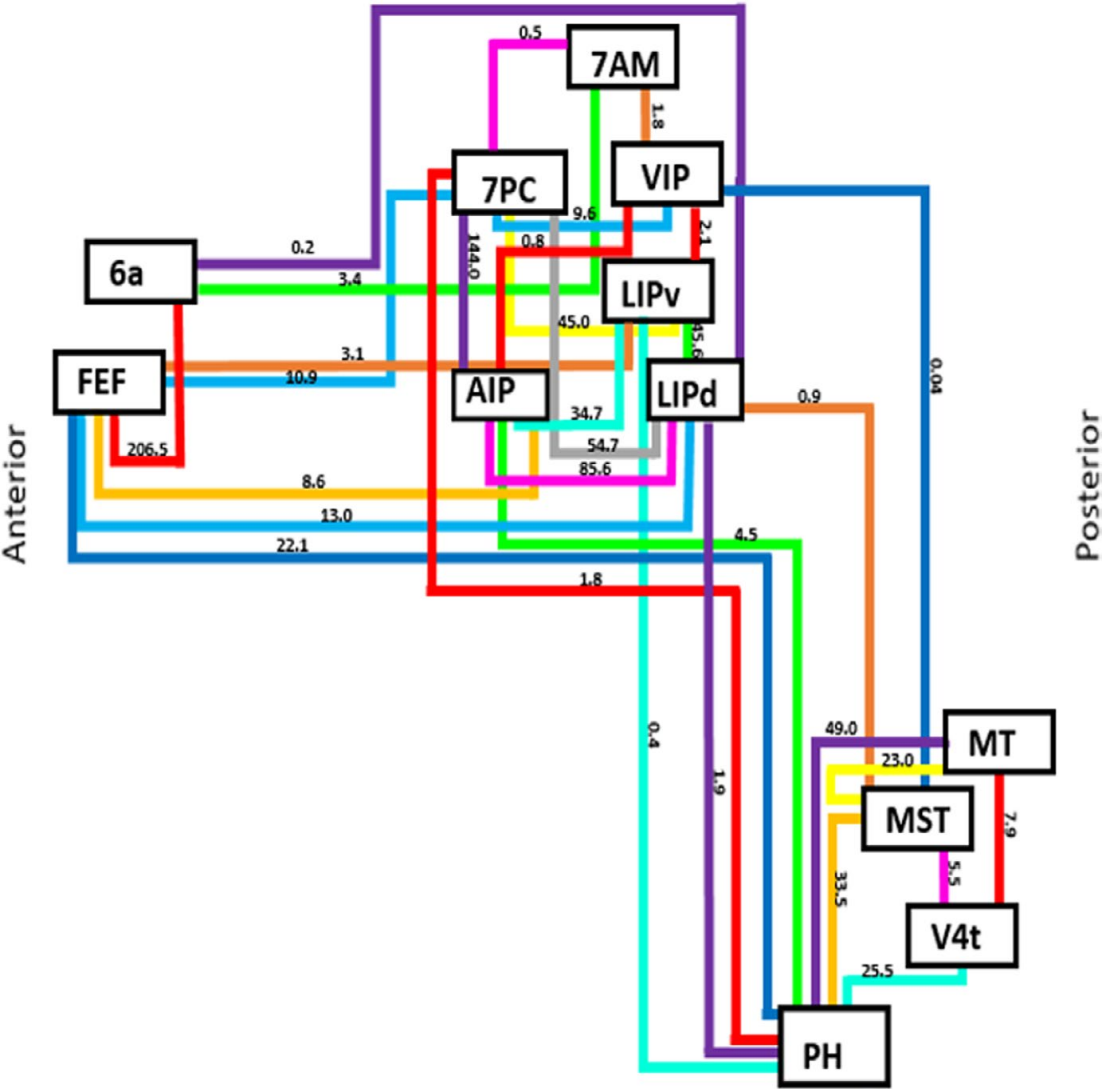
Simplified schematic of the white matter connections identified between individual parcellations of the dorsal attention network during fiber tracking analysis. Connections are labeled with their average strength measured across all 25 subjects used in this analysis

Connections between parietal areas 7PC, AIP, LIPd, LIPv, and VIP to the lateral occipital lobe (occipital areas PH and MST) were also identified. These fibers originate along the intraparietal sulcus and superior parietal lobule before coursing inferiorly to run within the deep white matter of the inferior parietal lobule. The fibers enter the subcortical white matter of the posterior temporal lobe, curving laterally to terminate in lateral the occipital cortex corresponding to areas PH and MST. In addition to these parietal–occipital connections, one fronto‐occipital connection was identified between areas FEF and PH. The connections of the DAN are summarized in Figure 4. Lines in this schematic represent individual connections of the DAN which are labeled with their average strength as measured across all 25 subjects included in this analysis.

## 4 DISCUSSION

In this study, we utilized meta‐analytic fMRI software and deterministic fiber tractography to construct a structural model of the DAN based on the cortical parcellation scheme previously published under the Human Connectome Project (Glasser et al., 2016). The DAN and VAN are known to mediate critical attentional processing in the cerebrum (Chica et al., 2013; Corbetta & Shulman, 2002). While the VAN is involved in reorienting attention from one object to another in the presence of unexpected, behaviorally relevant stimuli (Corbetta & Shulman, 2002; Hahn, Ross, & Stein, 2006), the DAN is responsible for the voluntary orientation of attention (Corbetta & Shulman, 2002; Ptak, 2012; Vossel, Geng, & Fink, 2014). The anatomic constituents of this network are discussed below.

### 4.1 The frontal lobe regions and the frontal eye fields

Cortical areas FEF and 6a overlap with the ALE in the frontal lobe. Area FEF represents the frontal eye field and is a well‐known component of the DAN (Corbetta & Shulman, 2002; Ozaki, 2011; Shulman et al., 2010; Spreng, Stevens, Chamberlain, Gilmore, & Schacter, 2010; Vossel, Weidner, Driver, Friston, & Fink, 2012). In contrast, area 6a is a newly described part of the cortex (Glasser et al., 2016). The ALE shows that these areas are activated bilaterally in the DAN which has been described in several fMRI‐related studies (Corbetta & Shulman, 2002; Joseph et al., 2015; Shulman et al., 2010). Both areas are interconnected by U‐shaped fibers and contribute to the fronto‐parietal projections of the SLF within the DAN.

Area FEF is located on the anterior half of the precentral gyrus, approximately half way down its length. It forms part of the floor of the precentral sulcus and extends anteriorly onto the posterior edge of the middle frontal gyrus. The area is known to be involved in intentional saccadic movements, as well as smooth eye pursuit when humans track a moving object (Fecteau & Munoz, 2006; Paus, 1996; Petit, Clark, Ingeholm, & Haxby, 1997; Pierrot‐Deseilligny, 1994; Pierrot‐Deseilligny, Gaymard, Muri, & Rivaud, 1997). In addition, while area 6a is relatively understudied, this region is located on the posterior–superior bank of the superior frontal sulcus and extends into the posterior most aspect of the superior frontal gyrus. It comprises part of the dorsal division of the premotor cortex (Glasser et al., 2016), which is involved in the preparation and planning of voluntary movement (Chouinard & Paus, 2006; Li, Chen, Guo, Gerfen, & Svoboda, 2015).

The precise nature of the relevance of area 6a in the DAN is not known, but the structural and functional connections between the FEF and area 6a suggest that the DAN is integrated within motor planning areas of the brain to maintain attention. Another possible explanation is that the DAN mediates attention during focused motor observation and learning (Wright et al., 2018).

### 4.2 The parietal lobe regions and the intraparietal sulcus

Similar to the FEF, the intraparietal sulcus and superior parietal lobule are also well‐established in the literature as part of the DAN (Asplund, Todd, Snyder, & Marois, 2010; Benedek et al., 2016; Corbetta & Shulman, 2002; Kraft, Sommer, Schmidt, & Brandt, 2011; Szczepanski et al., 2013). Regions 7AM, 7PC, AIP, LIPd, LIPv, and VIP overlap with the ALE in these parts of the cortex. The ALE constructed for the purposes of this study also demonstrates bilateral activation of the IPS, which has been demonstrated in several other studies (Corbetta & Shulman, 2002; Joseph et al., 2015; Shulman et al., 2010). The parcellations within the parietal lobe of the DAN display interconnectivity via U‐shaped fibers and connect to area FEF and 6a via the fronto‐parietal projections of the SLF. These regions also form the parieto‐occipital projections of the SLF that terminate in lateral visual cortex areas PH and MST. Several of these areas have been shown to be involved in the attentional processes related to eye movement, visuomotor activity, and visuospatial understanding.

Areas 7AM, 7PC, and VIP are the three parts of the DAN that are located predominantly within the superior parietal lobule. Area 7AM is located on the anterior superior surface and is involved in several types of information processing, including spatial, visual, and motor information (Wang et al., 2015). The anterior portion of area 7AM is also involved in attention‐related processed (Scheperjans et al., 2008). Area 7PC is located on the anterior inferior surface and extends into the posterior bank of the postcentral sulcus. Like area 7AM, area 7PC is also involved in several types of information processing, including spatial, visual, and motor information (Wang et al., 2015). Area VIP is located in the central most portion of the superior parietal lobule and is important in visual motion detection as well as the encoding of directional information (Galletti & Fattori, 2017; Grefkes & Fink, 2005).

The remaining areas identified as part of the DAN in the parietal lobe are all located in the cortical gray matter of the intraparietal sulcus, including areas AIP, LIPd, LIPv, and VIP. Area AIP is found on the anterior superior bank of the intraparietal sulcus and is involved in object recognition for grasping activity (Fogassi et al., 2001; Galletti & Fattori, 2018), as well as tactile shape‐processing and interpreting spatial orientation (Grefkes & Fink, 2005). Areas LIPd and LIPv are located on the superior banks of the intraparietal sulcus, with LIPv located superiorly to LIPd as it extends onto the inferior edge of the superior parietal lobule. This means area LIPd is actually located ventrally to area LIPv. Area LIPd has been implicated in the control of attention and eye movement related to saccade coordination and the mapping of contralateral three‐dimensional spaces (Grefkes & Fink, 2005). Area LIPv has also been implicated in the control of attention and eye movements (Grefkes & Fink, 2005), and is particularly important during visually guided reaching and pointing activities of the hand (Mars et al., 2011).

Given the role of the superior parietal lobule and intraparietal sulcus in visuomotor and visuospatial integration as well as attentional processing (Eckert et al., 2005; Husain & Nachev, 2007; Molenberghs, Mesulam, Peeters, & Vandenberghe, 2007; Wang et al., 2015; Wolpert, Goodbody, & Husain, 1998), it is unsurprising to us that parietal parcellations within these areas of cortex form part of the DAN. The regions highlighted here are likely important for the focused attention necessary during tool manipulation (Fogassi et al., 2001; Galletti & Fattori, 2018; Grefkes & Fink, 2005; Mars et al., 2011).

### 4.3 The occipital lobe regions and the lateral occipital cortex

The visual cortex, specifically the middle temporal area, has been shown to be a component of the DAN (Callejas, Shulman, & Corbetta, 2014; Corbetta & Shulman, 2011; Spreng et al., 2010; Umarova et al., 2010). We found that regions MST, MT, PH, and V4t overlap with the DAN ALE in the area of the lateral occipital cortex. Our ALE also showed bilateral activation of the visual cortex which is consistent with other studies (Joseph et al., 2015; Vossel et al., 2012). These regions display interconnectivity via U‐shaped fibers and connect to parietal and frontal areas via the parieto‐occipital and fronto‐occipital projections of the SLF.

Area PH is located in the anterior inferior lateral occipital lobe and is involved in the complex processing of place‐related information (Epstein, 2008; Grill‐Spector & Malach, 2004). Essentially, area PH encodes a representation of the local scene, implicating it in the formation of spatial maps, place encoding and place recognition (Epstein, 2008; Grill‐Spector & Malach, 2004). Area MST is located in the superior part of the lateral occipital lobe, below the angular gyrus of the inferior parietal lobule. This area receives direct, functional input from area MT and is responsible for the integration and analysis of global, visual motion and the perception of self‐motion (Britten, 2008). It is also involved in the execution and continuation of smooth pursuit eye movements, in coordination with the frontal eye fields (Born & Bradley, 2005; Ilg, 2008). Area MT is also located in the superior part of the lateral occipital lobe, inferior to the angular gyrus of the inferior parietal lobule. It is responsible for the integration of one‐dimensional visual signals into a two‐dimensional visual motion pattern, the segmentation of figure and background related to complex, moving stimuli, as well as the initiation of smooth pursuit eye movements in coordination with the frontal eye fields to aid in the focused attention on moving objects (Born & Bradley, 2005; Ilg, 2008). Area V4t is located in the central portion of the lateral occipital cortex. This area integrates information from both the ventral and dorsal streams and demonstrates a high level of activity in response to both motor and shape‐sensitive information, indicating its significance in the integration of object processing and global‐motion perception (Kolster, Peeters, & Orban, 2010).

While area MT has been shown to be active during smooth pursuit eye movements (Born & Bradley, 2005; Ilg, 2008), and, as a result, plays a role in the attention‐related tracking of moving objects, the roles of PH, MST, and V4t in attentional processing are not as well understood. Further studies are needed to characterize the precise role of these areas in the DAN. For example, regarding the functionality of area PH, there is the question of whether this part of the cortex encodes scenic information for use later by focusing one's attention on the immediate environment.

### 4.4 The strength of connections within the dorsal attention network

The strength of the connections identified between parcellations of the DAN is reported in Table 2. Two different values for strength were computed. This first represents the average strength as measured across all 25 subjects used in this analysis. The second represents the average strength when considering only those subjects demonstrating the connection when performing tractography. Based on these results, it is clear that the structural connectivity of the DAN varies to some degree between individuals. By presenting both sets of average connectional strengths, one can see how these connections vary in the network.

**Table 2:** Type and strength of connections within the dorsal attention network

Connection	Number of subjects	Average strength weighted by all subjects	Average strength weighted by identified subjects	Connection type
6a to 7AM	2/25 (8%)	3.4	42.5	SLF
6a to FEF	23/25 (92%)	206.5	224.5	U‐shaped fiber
6a to LIPd	2/25 (8%)	0.2	2.5	SLF
7AM to 7PC	4/25 (16%)	0.5	3.0	U‐shaped fiber
7AM to VIP	8/25 (32%)	1.8	5.6	U‐shaped fiber
7PC to AIP	21/25 (84%)	144.0	171.5	U‐shaped fiber
7PC to FEF	9/25 (36%)	10.9	30.3	SLF
7PC to LIPd	19/25 (76%)	54.7	71.9	U‐shaped fiber
7PC to LIPv	15/25 (60%)	45.0	75.0	U‐shaped fiber
7PC to PH	5/25 (20%)	1.8	9.0	SLF
7PC to VIP	13/25 (52%)	9.6	18.5	U‐shaped fiber
AIP to FEF	13/25 (52%)	8.6	16.5	SLF
AIP to LIPd	20/25 (80%)	85.6	107.1	U‐shaped fiber
AIP to LIPv	15/25 (60%)	34.7	57.8	U‐shaped fiber
AIP to PH	8/25 (32%)	4.5	14.1	SLF
AIP to VIP	3/25 (12%)	0.8	7.0	U‐shaped fiber
FEF to LIPd	15/25 (60%)	13.0	21.7	SLF
FEF to LIPv	5/25 (20%)	3.1	15.4	SLF
FEF to PH	12/25 (48%)	22.1	46.1	SLF
FEF to VIP	1/25 (4%)	0.9	23.0	SLF
LIPd to LIPv	12/25 (48%)	45.6	95.1	U‐shaped fiber
LIPd to MST	1/25 (4%)	0.9	23.0	SLF
LIPd to PH	5/25 (20%)	1.9	9.4	SLF
LIPd to VIP	9/25 (36%)	10.2	28.2	U‐shaped fiber
LIPv to PH	3/25 (12%)	0.4	3.7	SLF
LIPv to VIP	10/25 (40%)	2.1	5.2	U‐shaped fiber
MST to MT	17/25 (68%)	23.0	33.8	U‐shaped fiber
MST to PH	21/25 (84%)	33.5	39.9	U‐shaped fiber
MST to V4t	10/25 (40%)	5.5	13.8	U‐shaped fiber
MST to VIP	1/25 (4%)	0.04	1.0	SLF
MT to PH	24/25 (96%)	49.0	51.0	U‐shaped fiber
MT to V4t	12/25 (48%)	7.9	16.5	U‐shaped fiber
PH to V4t	20/25 (80%)	25.5	31.9	U‐shaped fiber

Abbreviation: SLF, superior longitudinal fasciculus.

It should also be noted that we did not set a threshold for the strength that might limit inclusion of certain connections within the network. For example, when evaluating the connection between MST and VIP via the SLF, the average strength across all 25 subjects was measured to be 0.04 versus 1.0 in the single subject identified as having this tract. If we had set a threshold of an average strength of 10.0 or set a threshold related to the frequency by which we saw the connection, that is, in at least 10 subjects, then we would not report this connection at all. In our view, this is incorrect. It more appropriate to say that the connection between MST and VIP is a relatively weak connection that occurs infrequently in the DAN, as opposed to saying no such connection exists. Despite not setting such a threshold, the strength and frequency of this connection raise an important question of whether it is critical to the functionality of the network. However, additional study is needed to answer this question.

### 4.5 Sensory modalities and the dorsal attention network

In this study, attentional experiments focusing on both visual and auditory modalities were included in the analysis. Some neuroscientists have found evidence for a modality‐specific DAN (Braga, Wilson, Sharp, Wise, & Leech, 2013). We recognize that different sensory modalities may recruit different areas of the brain when orienting attention. However, in this study, our aim was to identify and describe the major cortical inputs of the DAN using an established cortical parcellation scheme. Furthermore, some studies suggest that areas such as the frontal eye fields, intraparietal sulcus, and superior parietal lobule are active in attentional processing across different sensory modalities (Corbetta & Shulman, 2002; Rossi, Huang, Furtak, Belliveau, & Ahveninen, 2014). Future studies may explore the differences in DAN network topology during different attentional tasks associated with different sensory modalities.

## 5 CONCLUSIONS

We present a tractographic model of the dorsal attention network. This model comprises parcellations within the frontal, parietal, and occipital cortex which are principally linked through the superior longitudinal fasciculus. Future studies may refine this model with the ultimate goal of clinical application.

## CONFLICT OF INTEREST

None declared.

## Data Availability

The data that support the findings of this study are available from the corresponding author upon reasonable request.
